# Artificial Intelligence Language Models in Oncology: A Cross-Sectional Analysis of Published Studies

**DOI:** 10.7759/cureus.106983

**Published:** 2026-04-13

**Authors:** Nandi Edwards, Samy Kannout, Daniel Zhang, Henry C Y Wong, Jennifer Leigh, Charles B Simone, Ronald Chow

**Affiliations:** 1 Temerty Faculty of Medicine, University of Toronto, Toronto, CAN; 2 Siebel School of Computing and Data Science, University of Illinois Urbana-Champaign, Champaign, USA; 3 New York Proton Center, Memorial Sloan Kettering Cancer Center, New York, USA; 4 Centre for Evidence-Based Medicine, University of Oxford, Oxford, GBR

**Keywords:** artificial intelligence, cancer research, clinical evaluation, diagnostic accuracy, language models, medical informatics, meta-research, oncology research

## Abstract

Since its public release in November 2022, ChatGPT has been rapidly evaluated across medical disciplines, including oncology. However, the scope, methodological characteristics, and clinical focus of oncology-specific evaluations remain poorly characterized. We conducted a descriptive, cross-sectional meta-research analysis of oncology-related studies that explicitly evaluated ChatGPT, identified through Ovid Medline and Embase from database inception through December 25, 2025. Included studies were categorized by study design, oncology discipline, and artificial intelligence (AI) task, and results were summarized descriptively. A total of 1,325 oncology-related studies evaluating ChatGPT were identified, with publication volume increasing over time, including 128 (10%) in 2023, 540 (41%) in 2024, and 657 (50%) in 2025. Most studies were clinical studies (949, 72%), predominantly methodological or performance evaluation studies (658, 69% of clinical studies). Research activity was concentrated in general or multidisciplinary oncology (702, 53%) and radiation oncology (575, 43%), with limited representation in medical oncology (26, 2%) and basic science (22, 2%), and no identified studies in surgical oncology (0, 0%). ChatGPT was most frequently evaluated for diagnostic accuracy or classification tasks (760, 57%), followed by mixed AI tasks (371, 28%). Oncology-focused evaluations of ChatGPT are predominantly concentrated in methodological and performance-based study designs, with a strong emphasis on diagnostic applications and a limited range of oncology disciplines. These patterns indicate that current research has primarily focused on establishing model performance within structured evaluations, with comparatively less emphasis on broader clinical contexts. This study defines the current research landscape and provides a structured reference for interpreting how ChatGPT is being studied across oncology.

## Introduction and background

ChatGPT (OpenAI) is a large language model (LLM) developed by OpenAI and released in November 2022. It generates human-like text by learning patterns from large datasets, representing a shift beyond earlier rule-based artificial intelligence (AI) systems [1,2]. This capability has reduced barriers to use and accelerated the integration of LLMs into healthcare research and practice [3].

Within medicine, ChatGPT has been evaluated across several domains, including clinical documentation, evidence synthesis, clinical question answering, and medical education. These studies have suggested improvements in efficiency and accessibility in controlled settings [4,5]. However, important concerns remain. Hallucination, variable clinical accuracy, bias, privacy, and data security restrict its role to that of an assistive tool requiring human oversight, limiting its suitability for independent clinical decision-making [6].

Oncology represents a particularly relevant setting for evaluating ChatGPT. Cancer care involves large volumes of complex, heterogeneous, and longitudinal data that must be integrated to inform clinical decisions. Studies have explored applications in diagnostic reasoning, multidisciplinary treatment planning, clinical trial matching, report generation, and patient communication [7-10]. Most remain limited to proof-of-concept evaluations, leaving uncertainty regarding real-world clinical performance [3].

Understanding how technology is being evaluated provides insight into where evidence is concentrated and where gaps remain. To address this, we characterized oncology-focused ChatGPT studies by study design, oncology discipline, and AI task.

## Review

Study design, data sources, and classification

We searched Ovid MEDLINE and Embase using the key concepts of cancer and ChatGPT from database inception to December 25, 2025, with no language restrictions. Records were screened in duplicate using ChatGPT-assisted screening and an independent human reviewer. Studies were included if their abstracts reported on the use of ChatGPT in oncology.

Study design was categorized as basic, translational research, or clinical research. Clinical studies were further subclassified as methodological or evaluation studies, retrospective cohort studies, prospective cohort studies, survey studies, randomized controlled trials, systematic reviews or meta-analyses, or case reports or case series. The oncology discipline was classified as general or multidisciplinary oncology, radiation oncology, medical oncology, surgical oncology, or basic science. AI tasks were categorized as diagnostic accuracy or classification, benchmarking or performance evaluation, patient education, report generation, treatment planning, workflow support or automation, or mixed tasks.

Statistical analysis

All data were summarized descriptively.

Study characteristics

**Figure 1:**
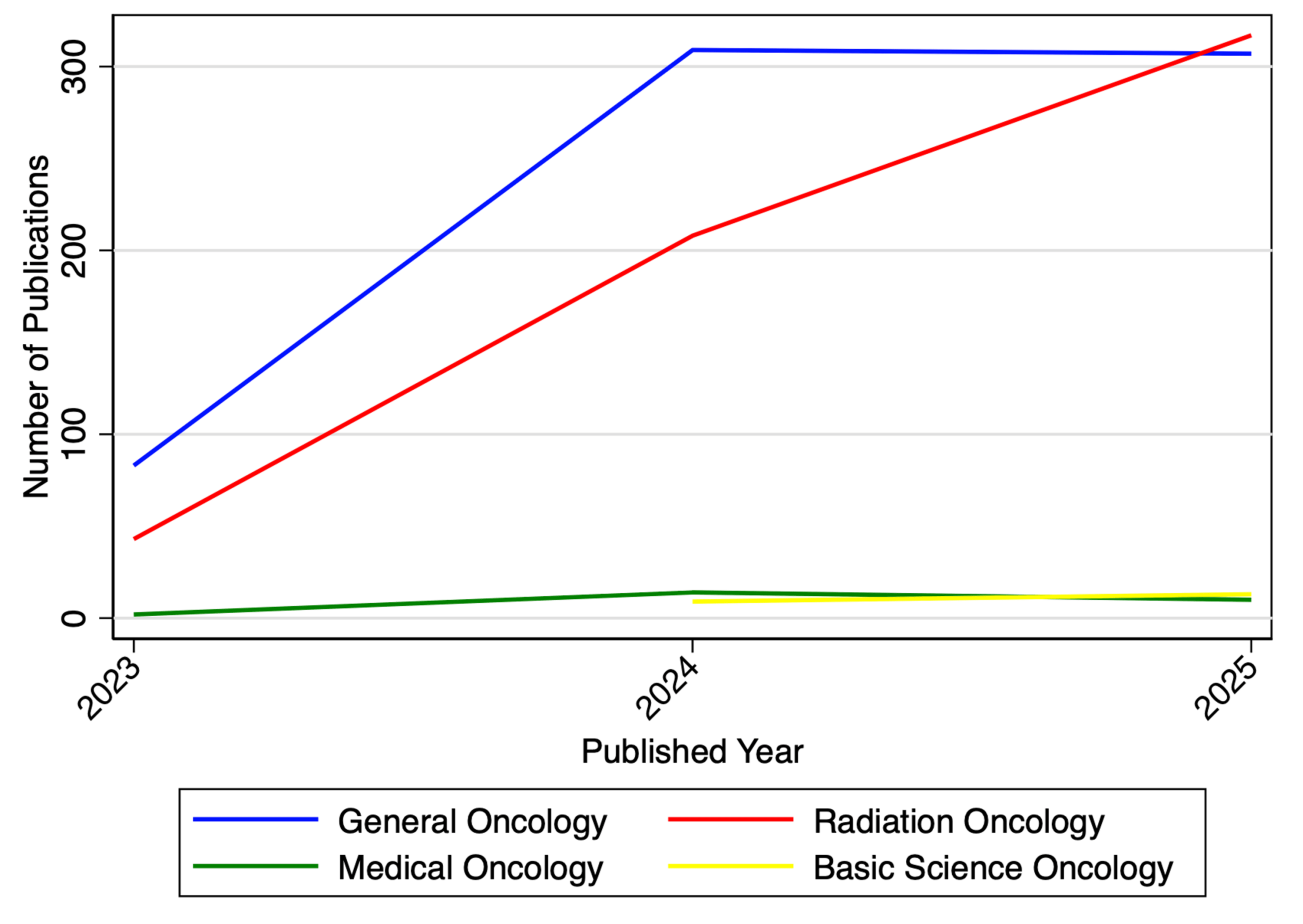
Number of studies evaluating ChatGPT in oncology published annually from 2023 to 2025.

A total of 1,325 oncology-related studies evaluating ChatGPT were identified. Publication volume increased over time, with 128 studies (10%) published in 2023, 540 (41%) in 2024, and 657 (50%) in 2025 (Figure 1).

Study design distribution

Most studies (949, 72%) were classified as clinical studies. Within this group, methodological or evaluation studies were the most common (658, 69% of clinical studies), followed by retrospective cohort studies (123, 13% of clinical studies). Smaller proportions included prospective cohort studies (42, 4% of clinical studies), survey studies (42, 4% of clinical studies; clinicians 21, 2% of clinical studies and patients 21, 2% of clinical studies), randomized controlled trials (39, 4% of clinical studies), systematic reviews or meta-analyses (32, 3% of clinical studies), and case reports or case series (13, 1% of clinical studies). Basic or translational research accounted for 376 studies (28%) (Table 1).

**Table 1:** Distribution of Published Studies (n = 1,325).

Characteristic	Distribution
Study design	
Clinical	949 (72%)
Methodological/evaluation study	658/949 (69%)
Retrospective cohort study	123/949 (13%)
Prospective cohort study	42/949 (4%)
Survey study	42/949 (4%)
Randomized controlled trial	39/949 (4%)
Systematic review/meta-analysis	32/949 (3%)
Case report/series	13/949 (1%)
Basic/translational	376 (28%)
Oncology discipline	
General/multidisciplinary	702 (53%)
Radiation oncology	575 (43%)
Medical oncology	26 (2%)
Basic science	22 (2%)
Surgical oncology	0 (0%)
Artificial intelligence task	
Diagnostic accuracy/classification	760 (57%)
Benchmarking/performance evaluation	62 (5%)
Patient education	57 (4%)
Report generation	53 (4%)
Treatment planning	16 (1%)
Workflow support/automation	6 (<1%)
Mixed artificial intelligence tasks	371 (28%)

Oncology discipline distribution

Research activity was concentrated in general or multidisciplinary oncology (702, 53%) and radiation oncology (575, 43%). Medical oncology (26, 2%) and basic science (22, 2%) were less frequently represented. No studies were identified in surgical oncology (0, 0%) (Table 1).

AI task distribution

ChatGPT was most frequently evaluated for diagnostic accuracy or classification (760, 57%). Additional studies examined benchmarking or performance evaluation (62, 5%), report generation (53, 4%), patient education (57, 4%), treatment planning (16, 1%), and workflow support or automation (6, <1%). Mixed-task studies accounted for 371 (28%) of the literature (Table 1).

Interpretation of findings

This meta-research study of published studies provides an overview of the current evidence base. Most studies assess ChatGPT's performance under controlled or simulated conditions, reflecting a focus on establishing baseline performance rather than real-world clinical use. This limits insight into how ChatGPT performs in practice, where decision-making is shaped by comorbidities, evolving disease trajectories, and patient preferences. Current evidence may therefore overestimate performance relative to clinical settings [11].

Distribution across disciplines and tasks

Research activity has been concentrated in general or multidisciplinary oncology and radiation oncology, with a strong emphasis on diagnostic and classification tasks. These areas align with language-based evaluation and existing digital infrastructure. In contrast, the absence of studies in surgical oncology likely reflects challenges in integrating LLMs into procedural workflows. This distribution suggests that research activity is influenced by feasibility, which may not align with areas of greatest clinical need [12-15].

Limitations

This study has limitations. As a descriptive analysis, it does not assess methodological quality, risk of bias, or clinical validity. The findings, therefore, reflect how ChatGPT has been studied rather than the strength of the evidence. In addition, while this analysis focuses on ChatGPT, other LLMs are increasingly being evaluated, and the research landscape is likely to evolve, which may affect the generalizability of these findings over time.

## Conclusions

In summary, oncology-focused ChatGPT research is predominantly concentrated in methodological and performance-based evaluations, with a strong emphasis on diagnostic tasks (760, 57%) and research activity concentrated in general or multidisciplinary (702, 53%) and radiation oncology (575, 43%). Surgical oncology remains unrepresented (0, 0%). These patterns indicate that current research has primarily focused on establishing model performance within structured evaluations, with comparatively less emphasis on broader clinical contexts and applications.

By consolidating how ChatGPT is currently being studied across oncology, this study defines the present research landscape and provides a structured reference for interpreting emerging evidence as the field continues to evolve.
